# Complete Genome Sequence of the Edible Filamentous Cyanobacterium Arthrospira platensis NIES-39, Based on Long-Read Sequencing

**DOI:** 10.1128/mra.01139-22

**Published:** 2022-12-20

**Authors:** Hideaki Shiraishi, Haruka Nishida

**Affiliations:** a Graduate School of Biostudies, Kyoto University, Kyoto, Japan; University of Delaware

## Abstract

Arthrospira platensis is a filamentous cyanobacterium that is cultivated and used worldwide as a source of food and food additives. Here, we report the complete genome sequence (6,818,916 bp) of A. platensis NIES-39, one of the model strains of A. platensis, providing an improved reference genome sequence for this strain.

## ANNOUNCEMENT

Arthrospira platensis is an edible cyanobacterium that is also known as spirulina (1, 2). NIES-39 is one of the model strains, and its draft genome sequence has been reported (3). However, the genome consists of 18 contigs separated by gaps. To better understand this strain's genetic properties, we decided to determine its full-length genome sequence.

The strain was obtained in 2008 from the Microbial Culture Collection at the National Institute for Environmental Studies (MCC-NIES) (Tsukuba, Japan), where it was maintained as a live culture. We also maintained it as a live culture until 2016. Then, it was cryopreserved since the conditions for cryopreservation were found (4). Cells were propagated from a single trichome in modified SOT medium (5, 6) and ground in liquid nitrogen using a mortar and pestle. DNA was extracted with phenol and purified twice by CsCl density gradient centrifugation (7, 8). The DNA was further purified using 0.87 volume of ProNex Size-Selective Chemistry reagent (Promega). The purified DNA was sonicated in a g-TUBE (Covaris) to obtain 10- to 20-kb fragments. A library was prepared using the SMRTbell Express template preparation kit v.2.0 (Pacific Biosciences [PacBio]). Sequence data were obtained using the Sequel IIe system (PacBio) and imported into SMRT Link v.10.1.0.119528 to obtain high-fidelity (HiFi) reads with quality values of >20 or 99% accuracy, which yielded 33,140 reads. Using Filtlong v.0.2.0 (https://github.com/rrwick/Filtlong), HiFi reads of >1,000 bp were selected, and the worst 10% of reads were discarded (parameters: --min_length 1000 --keep_percent 90). The resultant 29,202 reads (average length, 7,669 bp; *N*_50_, 8,626 bp) were used to assemble contigs with Flye v.2.9 (9), which yielded three contigs (3,451 kb, 2,846 kb, and 549 kb in length, with 33-fold coverage). Default parameters were used for all software unless otherwise noted.

**FIG 1:**
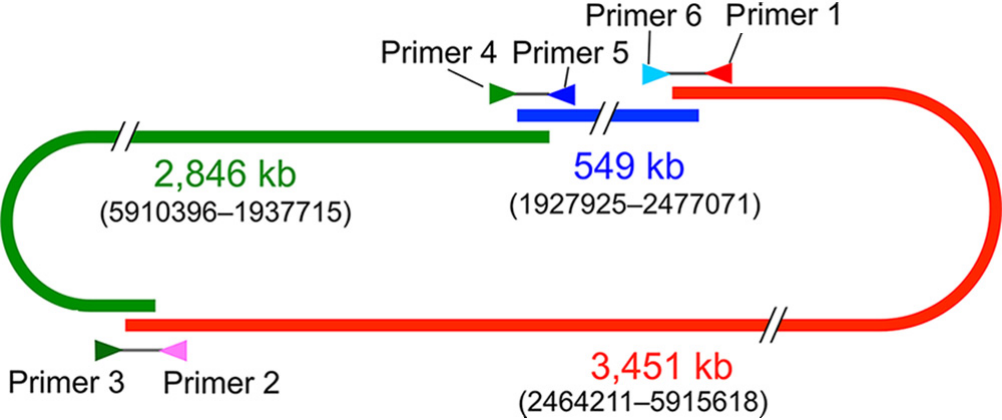
Schematic representation of junctional PCR. Numbers in parentheses show nucleotide positions in the finally determined full-length sequence.

All contigs contained members of dispersed repetitive sequences at both ends. To examine the relationships between the contigs, we synthesized six PCR primers that bind unique sequences near the termini of the contigs (Table 1 and Fig. 1). PCR amplification of the genomic DNA using KOD One PCR Master Mix (Toyobo) resulted in efficient amplification of contig junctions when three specific primer pairs were used, as summarized in Fig. 1. Analysis of the PCR-amplified DNAs with restriction enzymes indicated that, in the genomic DNA, each terminus of the contigs was connected to an adjacent one sharing the terminal sequence. To determine the precise nucleotide sequences of the contig junctions, we searched the FASTQ file for the HiFi reads containing the specific unique primer sequences used in the PCR mentioned above (Table 1). Through manual assembly of the extracted HiFi reads corresponding to each junction, the nucleotide sequences of the three contig junctions were unambiguously determined.

**TABLE 1:** List of PCR primers

Primer	Nucleotide sequence	Binding site^*a*^
Primer 1	5′-GCTTTGGGAGTGGTTGGGAAAATTTTTTGA-3′	2482240–2482211
Primer 2	5′-ATGATGTTGGGGATGTTTAGATAACTTTTG-3′	5904111–5904140
Primer 3	5′-ACTCCTAATAAACCACTAACTTTTCCTCCT-3′	5923425–5923396
Primer 4	5′-GCCAAGTGGTTAAGGCAGAGGATTGTGGTT-3′	1922043–1922072
Primer 5	5′-ACCACCATGATCATAACCATGATCATAGCC-3′	1945954–1945925
Primer 6	5′-GCCATCCAAGAATTTTTAGACGGTTCTGGT-3′	2461026–2461055

a Nucleotide positions in the finally determined full-length genome sequence are shown.

The genome of A. platensis NIES-39 was a circular DNA of 6,818,916 bp, with a GC content of 44.3%. Genome annotation using DFAST v.1.2.18 (10) with CyanoBase (11) as an optional database predicted 6,373 protein-coding sequences, 4 rRNA genes, and 48 tRNA genes. Among the newly found genes were the porphobilinogen deaminase gene (*hemC*), which is involved in heme biosynthesis, and a cluster of gas vesicle genes (*gvpK*-*gvpL*/*gvpF*-*gvpG*).

### Data availability.

The complete genome sequence has been deposited in GenBank under the accession number AP026945. The sequencing data can be found in the DDBJ Sequence Read Archive under the accession number DRR411675.
